# Mitochondrial responses to constant and cyclic hypoxia depend on the oxidized fuel in a hypoxia-tolerant marine bivalve *Crassostrea gigas*

**DOI:** 10.1038/s41598-024-60261-w

**Published:** 2024-04-26

**Authors:** Linda Adzigbli, Siriluck Ponsuksili, Inna Sokolova

**Affiliations:** 1 Institute for Farm Animal Biology, Institute of Genome Biology, Dummerstorf, Germany; 2 Department of Marine Biology, Institute for Biological Sciences, University of Rostock, Rostock, Germany; 3 Department of Maritime Systems, Interdisciplinary Faculty, University of Rostock, Rostock, Germany

**Keywords:** Mitochondrial substrate preference, Succinate, Electron transport system, Hypoxia-reoxygenation, Oxidative stress, Bioenergetics, Ecophysiology, Ecology, Ecophysiology

## Abstract

Sessile benthic organisms like oysters inhabit the intertidal zone, subject to alternating hypoxia and reoxygenation (H/R) episodes during tidal movements, impacting respiratory chain activities and metabolome compositions. We investigated the effects of constant severe hypoxia (90 min at ~ 0% O_2_ ) followed by 10 min reoxygenation, and cyclic hypoxia (5 cycles of 15 min at ~ 0% O_2_ and 10 min reoxygenation) on isolated mitochondria from the gill and the digestive gland of *Crassostrea gigas* respiring on pyruvate, palmitate, or succinate. Constant hypoxia suppressed oxidative phosphorylation (OXPHOS), particularly during Complex I-linked substrates oxidation. It had no effect on mitochondrial reactive oxygen species (ROS) efflux but increased fractional electron leak (FEL). In mitochondria oxidizing Complex I substrates, exposure to cyclic hypoxia prompted a significant drop after the first H/R cycle. In contrast, succinate-driven respiration only showed significant decline after the third to fifth H/R cycle. ROS efflux saw little change during cyclic hypoxia regardless of the oxidized substrate, but Complex I-driven FEL tended to increase with each subsequent H/R cycle. These observations suggest that succinate may serve as a beneficial stress fuel under H/R conditions, aiding in the post-hypoxic recovery of oysters by reducing oxidative stress and facilitating rapid ATP re-synthesis. The impacts of constant and cyclic hypoxia of similar duration on mitochondrial respiration and oxidative lesions in the proteins were comparable indicating that the mitochondrial damage is mostly determined by the lack of oxygen and mitochondrial depolarization. The ROS efflux in the mitochondria of oysters was minimally affected by oxygen fluctuations indicating that tight regulation of ROS production may contribute to robust mitochondrial phenotype of oysters and protect against H/R induced stress.

## Introduction

Aquatic ecosystems worldwide are experiencing a significant reduction in dissolved oxygen levels, a phenomenon known as hypoxia^1,2^. Coastal ecosystems are particularly vulnerable to hypoxia due to the combination of natural features (such as enrichment with organic matter, stratification and shallow depth) and anthropogenic nutrient pollution that stimulate bacterial respiration outstripping the oxygen input through photosynthesis, mixing and diffusion^1,2^. Depending on the local conditions, hypoxic episodes can last from several hours (during diurnal cycles of photosynthesis and respiration) to days and weeks in coastal dead zones^1–3^. Oxygen is essential for survival and development of most metazoans, making permanent dead zones incompatible with animal life and leading to a major loss of benthic biodiversity^4^. However, areas with periodic oxygen fluctuations (such as the intertidal zone or margins of the oxygen minimum zones) can support high diversity and biomass of benthic organisms adapted to hypoxia and reoxygenation stress^5–9^. Survival strategies during prolonged hypoxia center on energy conserving strategies such as the metabolic rate suppression and use of alternative anaerobic pathways with higher ATP yields^10–12^. Recovery from hypoxia presents additional challenges requiring rapid restoration of the homeostasis and avoidance of oxidative damage during reoxygenation^13–15^.

Mitochondria are a major target of hypoxia due to their key role in the oxygen-dependent ATP production and generation of reactive oxygen species (ROS). Under normal conditions, oxygen consumed by the mitochondria through the electron transfer system (ETS) generates the proton motive force driving synthesis of ATP in the process called oxidative phosphorylation (OXPHOS). Hypoxia disrupts ETS activity leading to lower rates of ATP production and elevated generation of ROS^16^. During reoxygenation, ROS efflux is further enhanced leading to mitochondrial damage^17^. The response of mitochondria to hypoxia-reoxygenation (H/R) stress differs between hypoxia-tolerant and intolerant species^15,18^. In hypoxia-intolerant species like terrestrial mammals and highly aerobic species of aquatic invertebrates, a suppression of ETS activity, mitochondrial depolarization and oxidative injury is observed after a single H/R cycle^19–21^. In contrast, in hypoxia-tolerant species like intertidal bivalves and some fishes, the ETS activity is stabilized or enhanced following H/R exposure^21–25^. Several adaptive mechanisms that might contribute to this metabolic resilience have been proposed including upregulation of ETS and antioxidant activity, enhanced protein quality control and suppression of ATP wastage^26–28^. Additionally, research findings have also revealed that changes in the intracellular mileu (such as shifts in the levels of metabolic intermediates) might contribute to the mitochondrial stress responses due to the use of alternative fuels for mitochondrial respiration^29,30^. Despite these advancements, our understanding of the mitochondrial mechanisms that contribute to the tolerance of animals to H/R stress remains limited. Critical questions remain unanswered, including the influence of the frequency and duration of hypoxic stress on mitochondrial damage and resilience, as well as the implications of alternative mitochondrial substrates on stress-induced changes in mitochondrial bioenergetics and ROS generation.

To fill these gaps in our knowledge, we conducted a study on the mitochondrial responses to varying H/R regimes in the Pacific oyster *Crassostrea (Magallana) gigas* Thunberg 1793, a marine bivalve known for its exceptional tolerance to hypoxia. *C. gigas* is a common intertidal species native to the Pacific coast of Asia and a successful invader in the intertidal zones worldwide, partially due to its high tolerance to abiotic stressors including oxygen fluctuations^31^. This makes the Pacific oyster an excellent model species to study mitochondrial flexibility under H/R stress. We focused on the intrinsic mechanisms of mitochondrial responses to different H/R regimes (constant and fluctuating hypoxia) using mitochondria isolated from two metabolically important organs of oysters—the gill and the digestive gland. Both organs are involved in energy metabolism with the gill responsible for oxygen uptake and filter-feeding^26,32^, and the digestive gland—for digestion and energy storage^33,34^. Here we studied how the mitochondria from the gill and the digestive gland of oysters responded to constant and intermittent hypoxia of similar duration (~ 90–125 min) and examined the dependence of the mitochondrial responses to H/R stress on the type of substrate used to fuel the respiration. While the duration of hypoxic exposures in our experiment was shorter than typical coastal hypoxia events^2,3^, it's important to recognize that experiments with isolated mitochondria are limited by their viability window, necessitating shorter exposures. Nonetheless, the oxygen regime used in our in vitro studies remains relevant for situations involving short-term oxygen fluctuations, such as those induced by valve closure and gaping behavior commonly observed in bivalves^35–37^.

We hypothesized that the constant (~ 90 min) severe hypoxia followed by reoxygenation will be more damaging to the oyster mitochondria than the cyclic H/R stress of similar total duration. Drawing on earlier research demonstrating the potential of succinate as a recovery fuel in hypoxia-tolerant marine bivalves^14,27,38^, we hypothesized that the capacity for mitochondrial succinate oxidation will increase in response to H/R stress without generating excess ROS, thereby mitigating the negative effects associated with H/R stress. To test our hypotheses, we conducted mitochondrial assays measuring basal and ADP-stimulated oxygen consumption rates and ROS efflux in isolated mitochondria from the gills and digestive gland of *C. gigas* under normoxic conditions and two H/R regimes: constant severe hypoxia (90 min at ~ 0% O_2_) followed by 10 min of reoxygenation, and cyclic H/R stress comprising five cycles of 15 min severe hypoxia and 10 min of reoxygenation each. We used a fatty acid (palmitate) and a carboxylic acid (pyruvate) as Complex I substrates, and succinate as a Complex II substrate. To assess the extent of ROS-induced damage to the mitochondria, we measured protein carbonyl accumulation in isolated gill mitochondria respiring on different substrates after constant and cyclic hypoxia exposures. Our findings offer insight into the mitochondrial mechanisms by which oysters adapt to H/R stress, potentially informing the development of new strategies for mitigating the negative effects of such stress.

## Materials and methods

### Chemicals

All chemicals were purchased from Fisher Scientific (Schwerte, Germany), Sigma Aldrich (Munich, Germany), or Carl Roth (Karlsruhe, Germany) and were of analytical grade or higher.

### Animals

Adult Pacific oysters *C. gigas* collected from the island of Sylt in the German Wadden Sea were transported within 24 h of collection to the University of Rostock. On arrival, oysters were acclimated for 3–6 weeks at 15 ± 1 °C temperature and 32 ± 1 (practical salinity units) salinity in aerated natural Baltic Sea water adjusted to a salinity of 32 with Instant Ocean sea salt (Aquarium Systems, Sarrebourg, France). The specified salinity and temperature conditions were representative of the habitat conditions during the time of collection. The oysters were fed ad libitum with a commercial algal blend (DT’s Live Marine Phytoplankton, CoralSands, Wiesbaden, Germany) according to the manufacturer’s recommendations.

### Mitochondrial isolation

**Figure 1:**
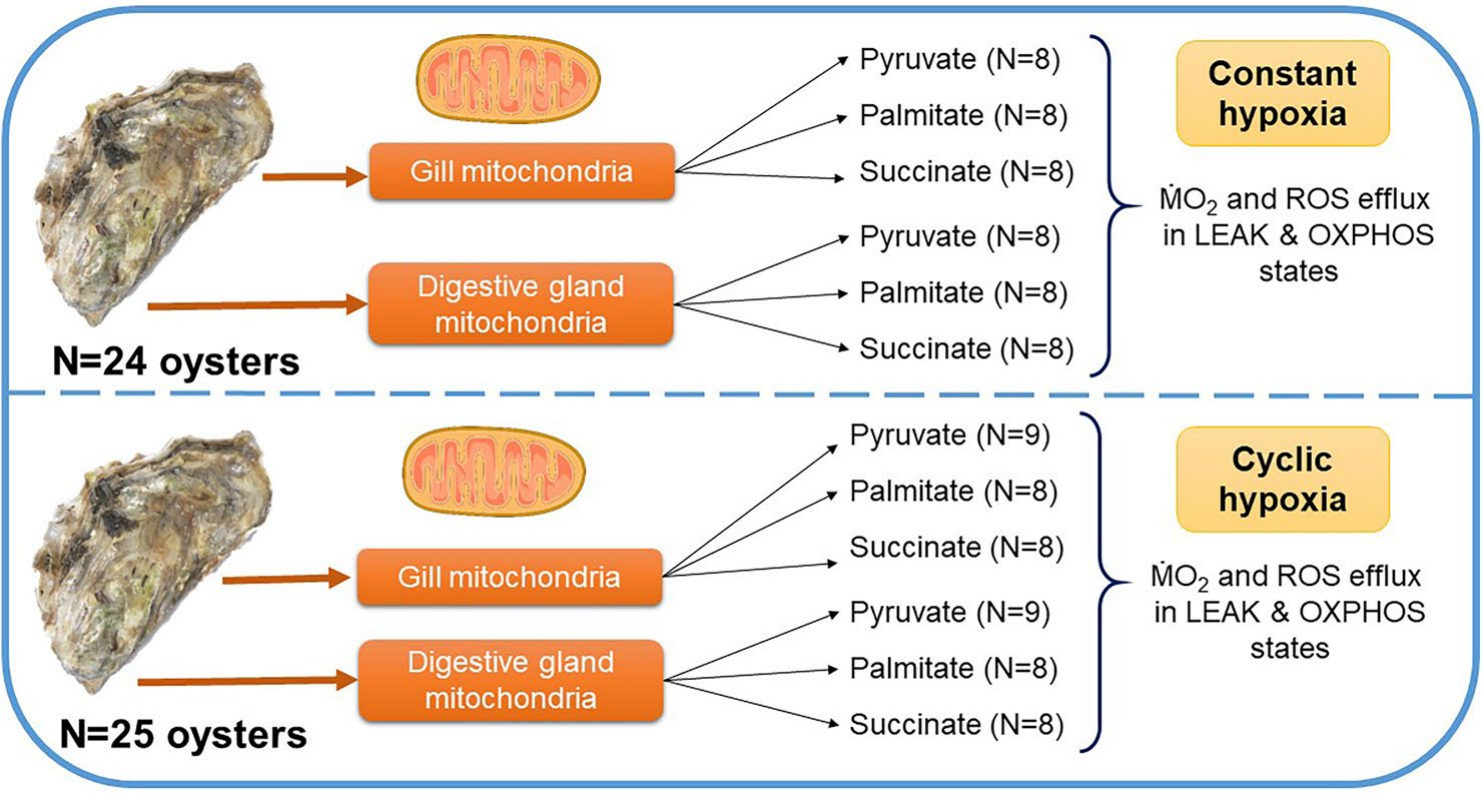
Schematic representation of the experimental design of the study.

Mitochondria isolates were obtained from the gill and digestive gland tissues of oysters. For each substrate and oxygen regime, mitochondria were isolated from the gill and the digestive gland of 8–9 oysters (Fig. 1). Overall, 24 and 25 oysters were used in the constant and cyclic hypoxia experiments, respectively, yielding a total of 98 mitochondrial isolates. Mitochondria from individual oysters were isolated as described elsewhere^38^ in an isolation medium (30 mmol l^−1^ 2-[4-(2-hydroxyethyl)piperazin-1-yl]ethanesulfonic acid (HEPES) pH 7.5, 100 mmol l^−1^ sucrose, 100 mmol l^−1^ NaCl, 200 mmol l^−1^ KCl, 8 mmol l^−1^ ethylene glycol-bis(2-aminoethylether)-N,N,N′,N′-tetraacetic acid, 1 mmol l^−1^ phenylmethylsulfonyl fluoride, 50 μg l^−1^ aprotinin). Mitochondrial pellets were resuspended in an ice-cold assay medium (30 mmol l^−1^ HEPES pH 7.2, 390 mmol l^−1^ sucrose, 10 mmol l^−1^ glucose, 130 mmol l^−1^ KCl, 10 mmol l^−1^ NaCl, 1 mmol l^−1^ MgCl_2_, 10 mmol l^−1^ KH_2_PO_4_ and 1% fatty acid free bovine serum albumin, BSA).

### Mitochondrial respiration indices and ROS measurements

Oxygen consumption rate (ṀO_2_) and hydrogen peroxide (H_2_O_2_) efflux rate were measured in isolated mitochondria at 15 °C using a high resolution Oxygraph 2-k respirometer (Oroboros, Innsbruck, Austria) with integrated DatLab 6 software. The H_2_O_2_ efflux rate is determined by the balance between the mitochondrial H_2_O_2_ production and consumption^39^ and referred to as ROS efflux. ṀO_2_ was measured using a Clark-type electrode and H_2_O_2_ with a Fluorescence-Sensor Green, both integrated with the Oxygraph-2k. Detailed procedures for the Oxygraph-2k calibration and substrate–uncoupler–inhibitor titration for measuring mitochondrial respiration and ROS efflux are outlined in^38^. Saturating concentrations of the respective substrates were used: (1) 10 μmol l^−1^ palmitoyl-dl-carnitine, (2) 5 mmol l^−1^ pyruvate with 2 mmol l^−1^ malate to spark respiration, (3) 10 mmol l^−1^ succinate. Baseline (LEAK) respiration (indicative of the rate of proton leak) was determined as the respiration of non-phosphorylating mitochondria in the presence of saturating concentrations of substrates. LEAK respiration was achieved either by addition of substrate without the addition of ADP (State II, before H/R) or by addition of an F_O_, F_1_ ATPase inhibitor oligomycin (State IV, after H/R). Pilot studies showed that the difference between the LEAK rate of the oyster mitochondria in State II and State IV is below 5% (data not shown). OXPHOS rate (reflective of ATP synthesis capacity) was determined as the rate of ADP-stimulated mitochondrial respiration.

### Hypoxia and reoxygenation exposures

Both constant and cyclic hypoxia exposures were conducted as separate experiments on isolated mitochondria in the respirometer chamber. After the addition of substrates and ADP to stimulate OXPHOS (State III respiration), the mitochondria were allowed to respire until all oxygen in the chamber was exhausted achieving severe hypoxia near anoxia (∼0% O_2_). For constant hypoxia, the period of severe hypoxia was maintained for 90 min, after which the oxygen tension was raised to ∼80% of air saturation and the mitochondria were allowed to recover for 10 min. For cyclic hypoxia, severe hypoxia was maintained for 15 min, after which the oxygen tension was raised to ∼50–80% of air saturation for 10 min. The mitochondria were then again allowed to exhaust all oxygen in the chamber and underwent another 15 min of severe hypoxia. The hypoxia and reoxygenation process was conducted for five consecutive cycles, and the last reoxygenation period was maintained for 10 min. Pilot experiments showed that there was no ADP limitation throughout the entire exposure period to the constant or cyclic hypoxia (data not shown). After the last reoxygenation cycle, 2.5 μmol l^−1^ oligomycin was added to the chamber to inhibit mitochondrial F_O_, F_1_-ATPase and measure LEAK respiration. This experimental design resulted in two values of respiration and ROS efflux measured in the LEAK state before the first and after the last H/R cycle. For the OXPHOS respiration and ROS efflux, two values (before and after H/R) were obtained in the constant hypoxia exposure, and six values (prior to hypoxia exposure and after each of the five cycles of H/R) were measured in the cyclic hypoxia exposures. Additionally, to ensure that the effects observed from the experiments were due to H/R stress and not the loss of mitochondria viability over time, parallel measurements were conducted in the mitochondria maintained for the same duration of time under normoxic conditions. The result revealed no difference in mitochondrial functional parameters of mitochondria maintained for ~ 125 min under normoxia (data not shown) eliminating the loss of mitochondrial viability over time under normoxic conditions.

Protein concentration was measured in the mitochondrial suspensions using the Bradford assay (Bio-Rad, Hercules, CA, USA) with BSA as a standard and corrected for the BSA content of the resuspension media. Respiration rates were expressed in nmol O_2_ min^−1^ mg^−1^ protein, and H_2_O_2_ efflux in nmol H_2_O_2_ min^−1^ mg^−1^ protein. The respiratory control ratio (RCR) was calculated as a ratio of OXPHOS to LEAK respiration rates^40^. The fractional electron leak (FEL) rate was determined by dividing the H_2_O_2_ efflux rate by the oxygen consumption rate in the same mitochondrial isolate.

### Measurement of protein carbonyls

Concentrations of carbonyls (as a proxy for oxidative damage of proteins) were determined by enzyme-linked immunosorbent assay (ELISA) in gill mitochondria only. After the mitochondrial assays were completed, mitochondria suspensions were collected from the control (normoxic) conditions and after both constant and cyclic hypoxia stress and stored at − 80 °C. For cyclic hypoxia, the suspensions were collected after the third H/R cycle to ensure the overall duration similar to the constant hypoxia exposures. Samples were lysed and diluted to 10 µg ml^−1^ protein with phosphate buffered saline (PBS) solution. ELISA protocol was adapted from ^41^ with modifications. Carbonyl standards were prepared by mixing varying amounts of oxidized BSA solution (IgG-free BSA dissolved in H_2_O_2_) and reduced BSA solution (IgG-free BSA dissolved in PBS) to a protein content of 10 µg ml^−1^. Carbonyl concentrations in the oxidized BSA were determined spectrophotometrically. Aliquots (100 μl) of samples or standards were placed into ELISA microwell plates and incubated overnight at 4 °C. Plates were washed three times with 250 μl of PBS. 100 μl solution of 5 mM 2, 4-dinitrophenylhydrazine was added to each well and incubated in the dark for 45 min at room temperature. After incubation, the plate was washed five times with PBS: ethanol (1:1 v:v) mixture and twice with PBS. The plate was blocked with 200 μl of 1 mg ml^−1^ BSA solution for 2 h at room temperature and washed thrice with 0.05% Tween solution. The primary anti-DNP antibody (mouse monoclonal, Sigma Aldrich MAB2223) was incubated for 1 h. After three washes with 0.05% Tween, the plates were incubated with a secondary anti-mouse antibody (Abcam) for 1 h at room temperature. The plate was washed five times with 0.05% Tween solution. 100 μl TMB/E ultra-sensitive blue Horseradish Peroxidase substrate was added to each well and upon color development (10 min), 100 μl 2 M sulfuric acid was added to stop the reaction. The absorbance was read at 450 nm. For protein carbonyls, sample size was 4–6 per substrate and hypoxia exposure group, with each biological replicate representing a separate mitochondrial isolate.

### Statistics

We conducted a normality test on the raw data using the Shapiro–Wilk Test and detected outliers with the Box and Whiskers plot using IBM^®^ SPSS^®^ Statistics ver. 22.0.0.0 (IBM Corp., Armonk, NY, USA). Data points were considered outliers if they fell outside 1.5 times the interquartile range and removed from the final analysis. Negative values in ROS efflux measurements, typically occurring during late cycles of cyclic H/R due to low mitochondrial activity, were removed as physiologically unfeasible. Final sample sizes are noted in the figure legends. To evaluate the interactive effect of the hypoxic state and respiratory substrate on mitochondrial oxygen consumption, ROS efflux, FEL and protein carbonyl accumulation, we used a repeated measures two-way general linearized ANOVA model with hypoxic state as a within-subject factor and respiratory substrate as a between-subject factor. The hypoxic state was treated as a within-subject factor since measurements of the mitochondrial traits under various oxygen treatments (normoxia and reoxygenation) were conducted in the same mitochondrial isolate. For constant hypoxia, the assumption of sphericity (equal variance) in the data was confirmed, leading to the recording of degrees of freedom and p-values for the within-subject effect based on the assumption of sphericity. However, for cyclic hypoxia stress, the assumption of sphericity was violated for some data sets. Therefore, degrees of freedom and p-values for the within-subject effect were calculated using the Greenhouse–Geisser correction. We used the Least Significant Difference (LSD) and Tukey’s honest significant differences (HSD) tests for planned comparisons of the group means. All statistical analyses were conducted using IBM^®^ SPSS^®^ Statistics ver. 22.0.0.0 (IBM Corp., Armonk, NY, USA) and GraphPad Prism v. 7.02 (GraphPad Software Inc., La Jolla, CA, USA) software. Differences were considered significant if the probability of type II error P was < 0.05. We have followed the recommendation of evidence-based language^42^ for describing our results. The following thresholds were used: P ≥ 0.05 (no evidence of effect), P = 0.049–0.011 (moderate evidence), P = 0.01–0.001 (strong evidence), P < 0.001 (very strong evidence of effect).

## Results

### Effects of constant hypoxia on mitochondrial functions

#### LEAK state

**Figure 2:**
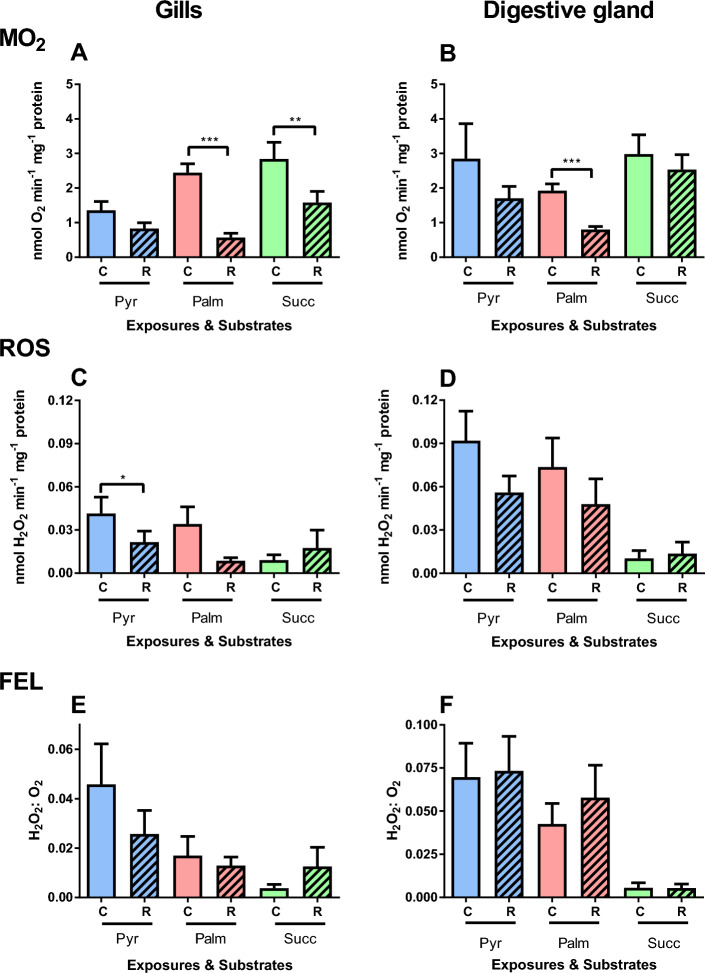
Effects of constant (~ 90 min) hypoxia and reoxygenation on LEAK respiration and ROS efflux of mitochondria isolated from the gills (**A**,**C**,**E**) or the digestive glands (**B**,**D**,**F**) of *C. gigas*. Substrates: Pyr—pyruvate, Palm—palmitate, and Succ—succinate. (**A**,**B**) Oxygen consumption rate, (**C**,**D**) ROS efflux rates, (**E**,**F**) FEL. Significant differences in a specific mitochondrial trait between normoxia (C, solid bars) and reoxygenation (R, striped bars) are denoted by asterisks (*P < 0.05, **P < 0.01, ***P < 0.001). N = 7 for pyruvate, and 8 for palmitate and succinate.

Our data showed strong evidence of the interactive effects of the substrate and constant (~ 90 min) hypoxia on the LEAK respiration in the gill mitochondria (Table 1). LEAK respiration was significantly suppressed in the gill mitochondria oxidizing palmitate and succinate but not affected in those oxidizing pyruvate (Fig. 2A). In the mitochondria from the digestive gland, there was moderate evidence for the effect of substrate on the LEAK respiration but no evidence for the effects of constant hypoxia or factor interactions (Table 1). LEAK respiration was suppressed after 90 min of hypoxia in the digestive gland mitochondria respiring on palmitate but not in those oxidizing pyruvate or succinate (Fig. 2B).

**Table 1:** Repeated measures ANOVA: Effects of constant hypoxia/reoxygenation and substrates on the respiration, ROS efflux and FEL in the mitochondria from the gill and the digestive gland (DG) of *C. gigas*.

Mitochondrial state	Factors		
	Oxygen regime	Substrate	Interactive effect
ṀO_2_			
LEAK_GILL_	**F**_**1,20**_** = 83.31, P < 0.001**	F_2,19_ = 3.02, P = 0.071	**F**_**2,20**_** = 8.29, P = 0.002**
LEAK_DG_	F_1,20_ = 2.95, P = 0.101	F_2,20_ = 5.05, P = **0.017**	F_2,20_ = 0.18, P = 0.833
OXPHOS_GILL_	**F**_**1,20**_** = 81.73, P < 0.001**	F_2,20_ = 11.91, P = **0.001**	**F**_**2,20**_** = 5.45, P = 0.013**
OXPHOS_DG_	**F**_**1,20**_** = 20.38, P < 0.001**	**F**_**2,20**_** = 4.99, P = 0.017**	F_2,20_ = 1.45, P = 0.257
ROS efflux			
LEAK_GILL_	**F**_**1,19**_** = 5.09, P = 0.036**	F_2,19_ = 1.30, P = 0.296	**F**_**2,19**_** = 3.63, P = 0.046**
LEAK_DG_	**F**_**1,19**_** = 6.96, P = 0.016**	F_2,19_ = 5.50, P = **0.013**	F_2,19_ = 2.54, P = 0.106
OXPHOS_GILL_	F_1,19_ = 0.43, P = 0.519	**F**_**2,19**_** = 6.04, P = 0.009**	F_2,19_ = 0.88, P = 0.430
OXPHOS_DG_	F_1,19_ = 0.000, P = 0.999	**F**_**2,19**_** = 6.54, P = 0.007**	F_2,19_ = 1.90, P = 0.177
FEL			
LEAK_GILL_	F_1,19_ = 1.86, P = 0.189	F_2,19_ = 2.93, P = 0.078	F_2,19_ = 2.26, P = 0.131
LEAK_DG_	F_1,19_ = 0.37, P = 0.552	**F**_**2,19**_** = 5.24, P = 0.015**	F_2,19_ = 0.10, P = 0.903
OXPHOS_GILL_	**F**_**1,20**_** = 9.95, P = 0.005**	**F**_**2,20**_** = 5.07, P = 0.017**	**F**_**2,20**_** = 4.97, P = 0.018**
OXPHOS_DG_	**F**_**1,20**_** = 17.67, P < 0.001**	**F**_**2,20**_** = 8.35, P = 0.002**	**F**_**2,20**_** = 4.08, P = 0.033**

Mitochondrial parameters were measured in the resting (LEAK) and actively phosphorylating (OXPHOS) states. F-values with the degrees of freedom for the effect and the error (in subscript) and P-values are given. Significant effects (P < 0.05) are highlighted in bold.

ROS efflux in the gill mitochondria in the LEAK state showed moderate interactive effect of constant hypoxia and substrate (Table 1). In the gill mitochondria respiring on pyruvate, ROS efflux in the LEAK state was suppressed after constant hypoxia (Fig. 2C). No evidence for change in ROS flux was found in the LEAK mitochondria respiring on palmitate or succinate (Fig. 2C). Moderate evidence was found for the effects of constant hypoxia and substrate on ROS efflux in the LEAK state mitochondria from the digestive gland (Table 1). ROS efflux rate and FEL tended to be lower in the digestive gland mitochondria respiring on succinate compared with those oxidizing pyruvate or palmitate (Fig. 2D). No evidence for the impact of prolonged hypoxia and reoxygenation was found for the ROS efflux or FEL, regardless of the substrate (Fig. 2D,F). The FEL in the gill mitochondria in the LEAK state remained unchanged after hypoxia exposure regardless of the substrate (Fig. 2E).

#### OXPHOS state

**Figure 3:**
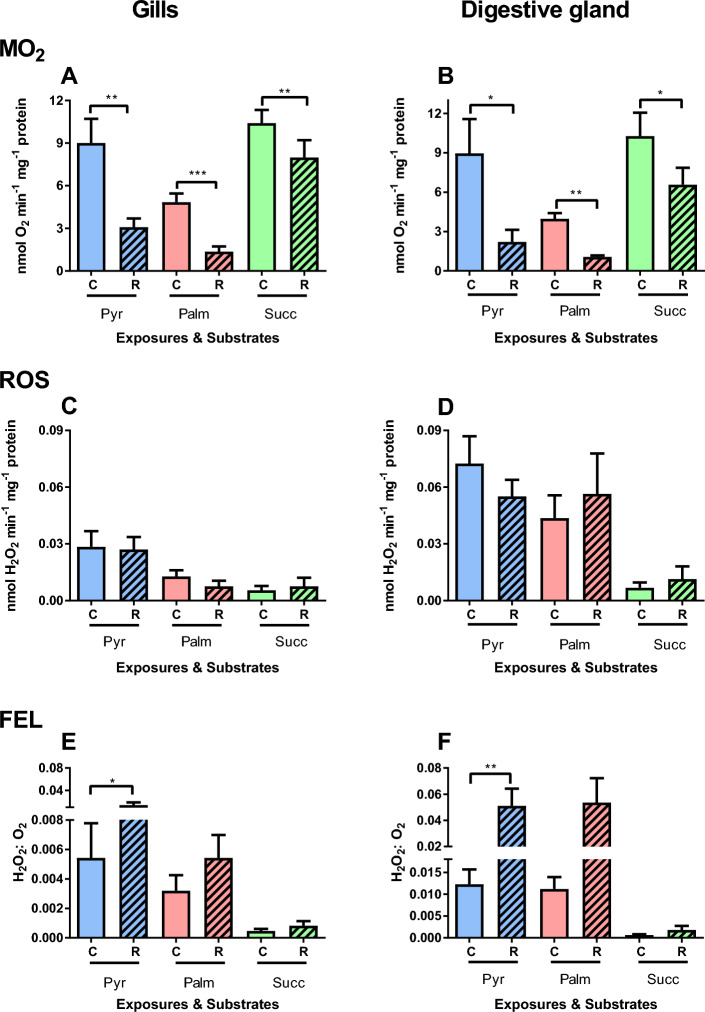
Effects of constant (~ 90 min) hypoxia and reoxygenation on OXPHOS respiration and ROS efflux of mitochondria isolated from the gills (**A**,**C**,**E**) or the digestive glands (**B**,**D**,**F**) of *C. gigas*. Substrates: Pyr—pyruvate, Palm—palmitate, and Succ—succinate. (**A**,**B**) Oxygen consumption rate, (**C**,**D**) ROS efflux rates, (**E**,**F**) FEL. Significant differences in a specific mitochondrial trait between normoxia (C, solid bars) and reoxygenation (R, striped bars) are denoted by asterisks (*P < 0.05, **P < 0.01, ***P < 0.001). N = 7 for pyruvate, and 8 for palmitate and succinate.

Moderate evidence for the interactive effects of the substrate and constant (~ 90 min) hypoxia was found for OXPHOS respiration of the gill mitochondria (Table 1). In the digestive gland mitochondria, OXPHOS respiration was significantly affected by constant hypoxia and substrate, but not by their interactions (Table 1). Generally, OXPHOS respiration was suppressed after prolonged hypoxia exposure in oyster mitochondria. The suppression was greater in the mitochondria respiring on Complex I substrates (pyruvate and palmitate) than in those oxidizing succinate (Fig. 3A,B).

ROS efflux in the actively phosphorylating (OXPHOS state) mitochondria from the gills and the digestive gland showed strong evidence of the effect of oxidized substrate but no effect of hypoxia exposure (Table 1, Fig. 3C,D). ROS efflux rates in the succinate oxidizing mitochondria were lower than in those respiring on pyruvate and palmitate (Fig. 3C,D). The FEL in the OXPHOS state mitochondria from the gill and the digestive gland showed moderate evidence of the interactive effects of hypoxia and substrate (Table 1). This reflected a major increase in the FEL after reoxygenation in the gill and the digestive gland mitochondria energized by pyruvate and (to a lesser degree) palmitate, which was not observed in the mitochondria oxidizing succinate (Fig. 3E,F).

### Effects of cyclic hypoxia on mitochondrial functions

#### LEAK state

**Figure 4:**
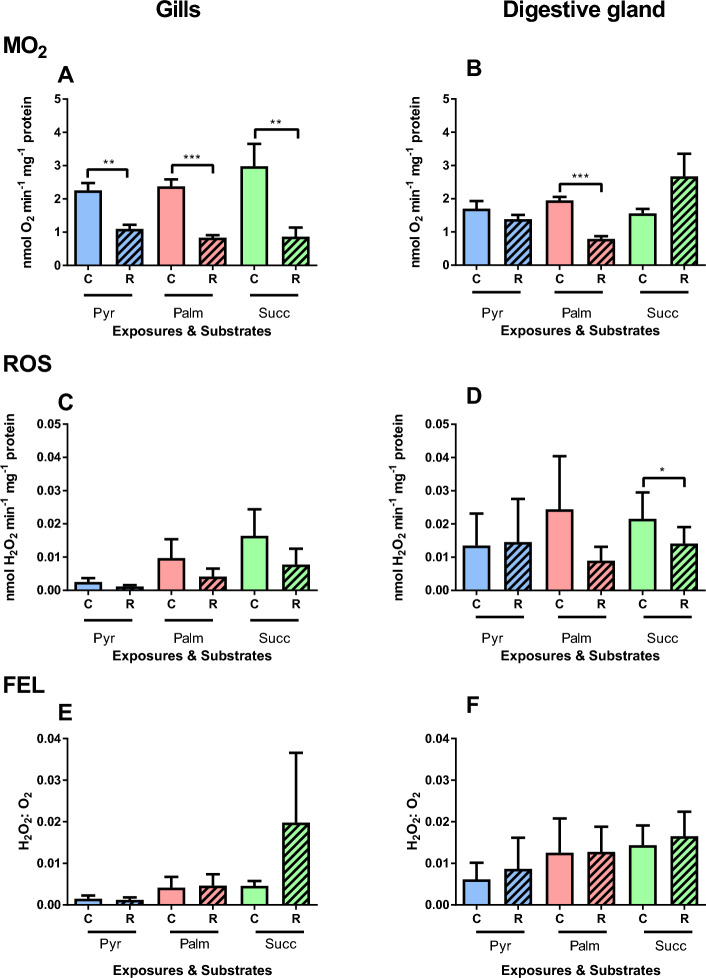
Effects of cyclic hypoxia on LEAK respiration and ROS efflux of mitochondria isolated from the gills (**A**,**C**,**E**) or the digestive glands (**B**,**D**,**F**) of *C. gigas*. Substrates: Pyr—pyruvate, Palm—palmitate, and Succ—succinate. Significant differences in a specific mitochondrial trait between normoxia (C, solid bars) and reoxygenation (R, striped bars) are denoted by asterisks (*P < 0.05, **P < 0.01, ***P < 0.001). N = 7–9 for pyruvate, 4–6 for palmitate, and 7–8 for succinate.

In the gill mitochondria, there was no evidence for the effects of substrates or factor interactions on LEAK respiration but very strong evidence of the effect of cyclic hypoxia (Table 2). This reflects a strong suppression in LEAK respiration observed after five H/R cycles with all studied substrates (Fig. 4A). In the mitochondria from the digestive gland, there was moderate evidence for the interactive effect of cyclic hypoxia and substrate (Table 2). In the digestive gland mitochondria, palmitate-driven LEAK respiration was significantly suppressed after H/R exposures whereas the pyruvate- and succinate-driven LEAK respiration did not change (Fig. 4B).

**Table 2:** Repeated measures ANOVA: Effects of cyclic hypoxia (5 cycles of 15 min anoxia followed by reoxygenation) and substrates on the respiration, ROS efflux and FEL in the mitochondria from the gill and the digestive gland (DG) of *C. gigas*.

Mitochondrial state	Factors		
	Oxygen regime	Substrate	Interactive effect
ṀO_2_			
LEAK_GILL_	**F**_**1,17**_** = 40.71, P < 0.001**	F_2,17_ = 0.28, P = 0.757	**F**_2,17_** = **1.29, P = 0.301
LEAK_DG_	**F**_**1,20**_** = 24.66, P < 0.001**	**F**_**2,20**_** = 5.42, P = 0.013**	**F**_**2,20**_** = 5.16, P = 0.016**
OXPHOS_GILL_	**F**_**1.5,25**_** = 20.85, P < 0.001**	**F**_**2,17**_** = 4.60, P = 0.025**	F_2.9,35_ = 1.17, P = 0.339
OXPHOS_DG_	**F**_**1.3,26**_** = 60.84, P < 0.001**	**F**_**2,20**_** = 8.07, P = 0.003**	**F**_**2.6,26**_** = 6.76, P = 0.002**
ROS efflux			
LEAK_GILL_	F_1,16_ = 2.29, P = 0.149	F_2,16_ = 1.47, P = 0.260	**F**_**2,16**_ = 0.42, P = 0.663
LEAK_DG_	F_1,20_ = 3.14, P = 0.092	F_2,20_ = 0.02, P = 0.979	F_2,20_ = 1.52, P = 0.243
OXPHOS_GILL_	F_1.5,24_ = 1.14, P = 0.323	F_2,16_ = 1.60, P = 0.232	F_3,24_ = 0.62, P = 0.610
OXPHOS_DG_	F_1.1,20.7_ = 1.24, P = 0.284	F_2,19_ = 0.11, P = 0.894	F_2.2,20.7_ = 0.41, P = 0.684
FEL			
LEAK_GILL_	F_1,16_ = 0.17, P = 0.690	F_3,16_ = 0.55, P = 0.655	F_3,16_ = 0.33, P = 0.806
LEAK_DG_	F_1,18_ = 0.23, P = 0.635	F_2,18_ = 0.91, P = 0.420	F_2,18_ = 0.31, P = 0.740
OXPHOS_GILL_	F_1.2,19.6_ = 1.34, P = 0.270	F_2,16_ = 2.66, P = 0.101	F_2.5,19.6_ = 1.18, P = 0.336
OXPHOS_DG_	F_1.1,21.4_ = 3.56, P = 0.069	F_2,19_ = 0.88, P = 0.431	F_2.2,21.4_ = 0.49, P = 0.639

Mitochondrial parameters were measured in the resting (LEAK) and actively phosphorylating (OXPHOS) states. F-values with the degrees of freedom for the effect and the error (in subscript) and P-values are given. Significant effects (P < 0.05) are highlighted in bold.

There was no evidence for the effect of the cyclic hypoxia, substrate, or factor interactions on ROS efflux or FEL in the LEAK state mitochondria from the gill or the digestive gland (Table 2). In the gill mitochondria, no change in the ROS efflux or FEL was found before and after the cyclic H/R stress (Fig. 4C,E). In the digestive gland mitochondria, ROS efflux rate in succinate-oxidizing mitochondria decreased after cyclic H/R stress, whereas no change was found in the mitochondria respiring on palmitate or pyruvate (Fig. 4D). No change in the FEL was found in the digestive gland mitochondria after cyclic H/R hypoxia (Fig. 4F).

#### OXPHOS state

**Figure 5:**
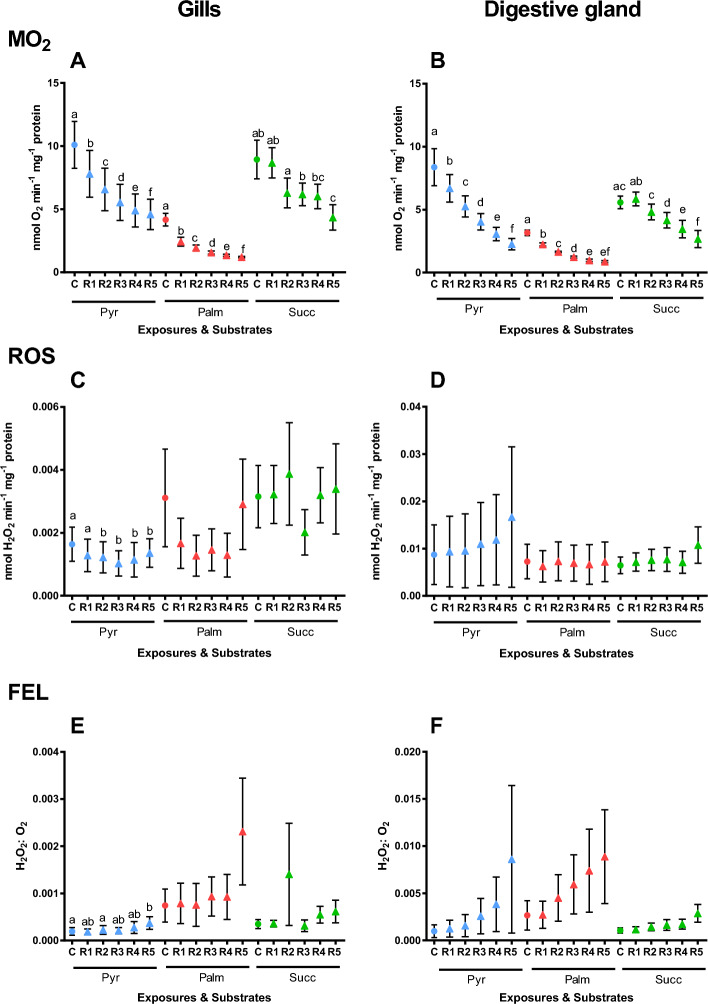
Effects of cyclic hypoxia on OXPHOS respiration and ROS efflux of mitochondria isolated from the gills (**A**,**C**,**E**) or the digestive glands (**B**,**D**,**F**) of *C. gigas*. Substrates: Pyr—pyruvate, Palm—palmitate, and Succ—succinate. Mitochondrial traits were measured under normoxia (control, C) and after sequential H/R cycles: —cycle 1, R2—cycle 2, R3—cycle 3, R4—cycle 4, R5—cycle 5 of H/R. Significant differences in a specific mitochondrial trait measured with the same substrate under different oxygen conditions (normoxia shown by circles and subsequent H/R cycles shown by triangles) are denoted by lowercase letters. Values that do not share a letter are significantly different (*P* < 0.05). Absence of letters indicate that no significant differences between normoxia and different H/R cycles were detected (*P* > 0.05). N = 7–9 for pyruvate, 4–6 for palmitate, and 7–8 for succinate.

In the gill mitochondria, there was moderate evidence for the effect of substrate and very strong evidence of the effect of cyclic hypoxia on OXPHOS respiration (Table 2). Generally, there was a gradual decline in OXPHOS respiration rate with each consequent H/R cycle. Notably, the decline in the OXPHOS respiration rate was more pronounced in the gill mitochondria respiring on pyruvate and palmitate where a significant drop was observed after the first H/R cycle through to the fifth H/R cycle (Fig. 5A). In the gill mitochondria oxidizing succinate, a decrease in OXPHOS respiration was observed after the fifth H/R cycle (Fig. 5A). In the digestive gland mitochondria, there was strong evidence for the interactive effect of substrate and cyclic hypoxia on OXPHOS respiration (Table 2). Similar to the gills, a decline in the OXPHOS respiration rate with NADH-linked substrate was observed after the first H/R cycle in the digestive gland mitochondria (Fig. 5B). A significant decline in succinate-driven OXPHOS respiration was observed after the third H/R cycle in the digestive gland mitochondria (Fig. 5B).

During OXPHOS respiration, there was no evidence for the effect of oxygen regime, substrate, or factor interaction on ROS efflux and FEL in the gill mitochondria (Table 2). In the gill mitochondria respiring on pyruvate, there was a significant decline in ROS efflux after the second H/R cycle and increase in FEL after the fifth cycle (Fig. 5C,E). ROS efflux and FEL of the gill mitochondria respiring on palmitate or succinate showed no evidence of change during cyclic hypoxia (Fig. 5C,E). There was no evidence of the effects of the effect of oxygen regime, substrate, or factor interaction on ROS efflux in the digestive gland mitochondria (Table 2; Fig. 5D), whereas FEL was significantly affected by cyclic hypoxia showing increasing trend with each consequent H/R cycle (Table 2; Fig. 4F).

#### Mitochondrial coupling

**Figure 6:**
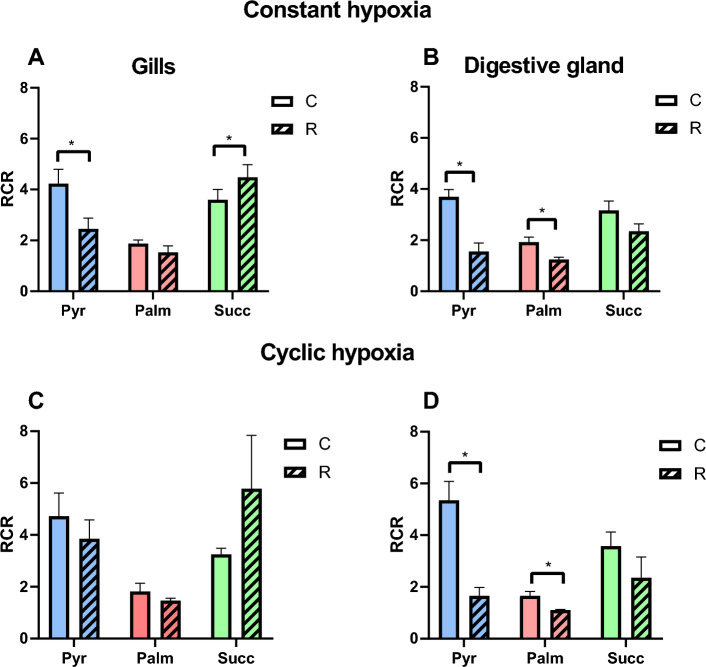
Effects of constant (**A**,**B**) and cyclic (**C**,**D**) hypoxia on respiratory control ratio (RCR) of mitochondria isolated from the gills (**A**,**C**) or the digestive glands (**B**,**D**) of *C. gigas*. Substrates: Pyr—pyruvate, Palm—palmitate, and Succ—succinate. Significant differences in a specific mitochondrial trait between normoxia (C, solid bars) and reoxygenation (R, striped bars) are denoted by asterisks (*P < 0.05, **P < 0.01, ***P < 0.001). N = 7–9 for pyruvate, 5–8 for palmitate, and 7–8 for succinate.

In the gill mitochondria, exposure to constant hypoxia suppressed mitochondrial RCR during pyruvate-driven oxidation and increased it under succinate-driven oxidation (Fig. 6A). In the digestive gland, constant hypoxia led to a decline in RCR of the mitochondria respiring on pyruvate and palmitate, but no change was found in those oxidizing succinate (Fig. 6B). A similar pattern was found in the mitochondria exposed to the cyclic hypoxia (Fig. 6C,D).

#### Oxidative damage

**Figure 7:**
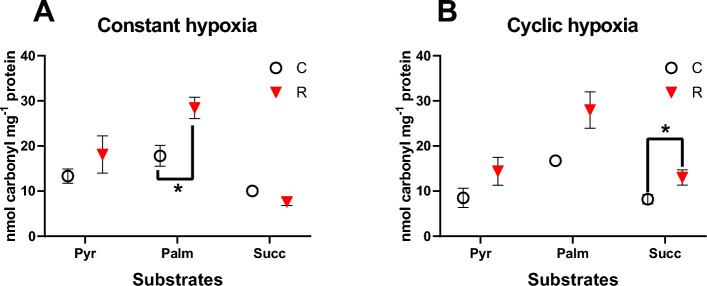
Effects of constant (**A**) and cyclic (**C**) hypoxia on protein carbonyl content of isolated gill mitochondria of *C. gigas* respiring on different substrates. Substrates: Pyr—pyruvate, Palm—palmitate, and Succ—succinate. Significant differences in a specific mitochondrial trait between normoxia (C, circles) and reoxygenation (R, triangles) are denoted by asterisks (*P < 0.05, **P < 0.01, ***P < 0.001). N = 4–6.

Constant and cyclic hypoxia led to an increase in the carbonyl content of proteins in isolated gill mitochondria under most experimental conditions (Table 3, Fig. 7). This increase was significant in palmitate-oxidizing mitochondria after constant hypoxia and in succinate-oxidizing mitochondria after cyclic H/R stress.

**Table 3:** Repeated measures ANOVA: Effects of the constant and cyclic hypoxia and reoxygenation and substrates on the protein carbonyl content of the mitochondria isolated from the gills of *C. gigas*.

	Oxygen regime	Substrates	Interactive effect
Constant hypoxia	**F**_**1,12**_** = 6.94, P = 0.022**	**F**_**1,12**_** = 10.74, P = 0.002**	**F**_**1,12**_** = 5.62, P = 0.019**
Cyclic hypoxia	**F**_**1,20**_** = 83.31, P** < **0.001**	F_1,20_ = 3.02, P = 0.071	**F**_**2,20**_** = 8.29, P = 0.002**

Significant values are in bold.

## Discussion

Pacific oysters are exceptionally stress-tolerant bivalves able to survive exposures to extreme temperature, salinity and oxygen fluctuations^43–45^. In oysters, exposure to severe hypoxia leads to metabolic rate suppression with simultaneous activation of anaerobic pathways for ATP generation and antioxidant activities^46^. One of the major consequences of anaerobic transition is alteration of the metabolome composition including changes in the concentrations of mitochondrial substrates^32,46^. During hypoxia, reduced intermediates like NADH and succinate accumulate and can drive mitochondrial ROS production during reoxygenation^30,47,48^. Our findings demonstrate that mitochondrial responses to H/R stress in a hypoxia-tolerant marine bivalve are modulated by the substrate oxidized by mitochondria. This is indicated by significant interactive effects of substrate and oxygen regime on mitochondrial oxygen consumption in both LEAK and OXPHOS states during constant hypoxia in the gills (Table 1) and during cyclic hypoxia in the digestive gland (Table 2). Notably, no evidence of the interactive effects of substrate and oxygen regime was found on the mitochondrial ROS efflux indicating than net ROS production during H/R stress is similar with Complex I (pyruvate and palmitate) and Complex II (succinate) substrates (Table 2; Figs. 4C,D, 5C,D). This finding is consistent with the earlier studies showing negligible ROS production due to the reverse electron flux in oysters^38^ in contrast to hypoxia-intolerant species such as some terrestrial mammals where succinate oxidation leads to a major increase of ROS production due to the reverse electron transport (RET)^30,49^.

Constant and cyclic hypoxia suppressed oxygen consumption in the mitochondria of *C. gigas*. The oyster mitochondria were considerably more susceptible to H/R-induced loss of respiration capacity during oxidation of Complex I substrates compared with the Complex II substrate. Thus, OXPHOS rate in the oyster mitochondria oxidizing pyruvate or palmitate declined by 63–70%, whereas those oxidizing succinate decreased by 24–31% after 90 min of constant hypoxia. Similarly, three H/R cycles (of comparable duration to the constant hypoxia in our present study) led to a 45–52% decrease in OXPHOS respiration fueled by pyruvate, 61–62% decline in palmitate-driven OXPHOS and only 25–30% decrease in the succinate-driven OXPHOS respiration. The decline in OXPHOS rate continued during cyclic hypoxia with the loss of 55–73%, 71–73%, and 51–52% of OXPHOS activity with pyruvate, palmitate and succinate, respectively, after five H/R cycles. Generally, palmitate oxidation generated the lowest respiratory flux under normoxia and the highest decline both under constant and cyclic hypoxia in oyster mitochondria. A similar observation was made in *C. gigas* mitochondria exposed to acute short-term (15 min) hypoxia^38^ suggesting the inability of oyster mitochondria to effectively utilize fatty acids especially during intermittent hypoxia. Studies of other marine mollusks also reveal their limited capacity in oxidizing fatty acids^50–52^.

Interestingly, the resting (LEAK) respiration with Complex I substrates was generally less susceptible to cyclic hypoxia than OXPHOS rate (Fig. 3C,D). As a result of this discrepancy, the mitochondrial coupling efficiency (RCR) decreased after cyclic hypoxia in the oyster mitochondria respiring on Complex I substrates. This was not observed during the succinate oxidation where the mitochondrial coupling efficiency increased or remained the same after H/R stress. Interestingly, our earlier study using a short-term (15 min) hypoxia did not detect any suppression of Complex I-dependent respiration in oyster mitochondria^38^. This indicates that increasing hypoxic duration leads to progressive inactivation of Complex I in oysters.

Complex I have been reported as the most vulnerable ETS complex to H/R stress^53,54^. Partial suppression of Complex I activity by H/R stress has been observed in hypoxia-intolerant^55,56^ and some hypoxia-tolerant species^57,58^. Depending on the species, the mechanisms of this suppression might include regulated inactivation (e.g. due to post-translational modification of Complex I proteins) and/or oxidative and nitrosative damage caused by H/R stress^55,56^. Suppression of Complex I activity can impair OXPHOS flux capacity that is predominantly controlled by ETS activity in bivalves^59–61^ and can explain the observed suppression in OXPHOS fueled by pyruvate and palmitate in oysters. However, suppression of Complex I activity might also serve as a protective mechanism preventing excessive ROS production during reoxygenation, since Complex I is one of the main sites of ROS generation^54,62^. Here, we observed that the impact of H/R on ROS efflux via Complex I-linked substrate oxidation was dependent on the mitochondrial activity state. Thus, in the resting (LEAK state) mitochondria respiring on Complex I-linked substrate, constant (90 min) hypoxia followed by reoxygenation suppressed ROS efflux by 34–47%. Under these conditions, the decrease in ROS generation was roughly proportional to the decline in LEAK respiration, hence the FEL rate did not change. Similarly, after five H/R cycles the change in ROS efflux rate of the LEAK state mitochondria respiring with pyruvate or palmitate were proportional to the changes in the oxygen consumption resulting in a relatively stable FEL. In contrast, pyruvate- and palmitate-driven ROS efflux remained unchanged or slightly increased in OXPHOS state mitochondria after constant hypoxia leading to an increased FEL. A similar increasing trend of FEL was found during sequential H/R cycles in OXPHOS state mitochondria energized by pyruvate or palmitate. Elevated FEL was associated with higher levels of the oxidative damage (indicated by accumulation of protein carbonyls) in oyster mitochondria respiring on Complex I substrates, particularly during the oxidation of palmitate.

Unlike Complex I substrates, oxidation of Complex II substrate (succinate) was more robust to hypoxia and reoxygenation in oyster mitochondria. Thus, a decline in succinate-driven respiration was 2–3 times lower than that observed during oxidation of Complex I substrates. Previous studies in oysters exposed to H/R stress in vivo revealed stimulation of succinate-driven LEAK and OXPHOS respiration in the gill mitochondria^27,63^. In vitro exposure of mussel and oyster mitochondria to short-term (15 min) hypoxia and subsequent reoxygenation also stimulated rather than suppressed Complex II-driven respiration^14,38^. In *Drosophila,* exposure to high temperatures switched the mitochondrial substrate preference from Complex I substrates to succinate thus maintaining respiration despite the heat-induced decrease in Complex I activity^64,65^. Taken together, these findings indicate that succinate might be a preferred mitochondrial fuel under stress conditions like H/R exposures or heat stress^66^. Furthermore, succinate oxidation acts as a regulatory and compensatory mechanism for maintaining of the mitochondrial membrane potential, ATP synthesis and adenylate pool^67–69^. In marine bivalves including oysters, succinate is the major anaerobic end product that accumulates in high concentrations during hypoxia^25,32,46,70,71^. Thus, high capacity for succinate oxidation might be adaptive during post-hypoxic recovery in oysters helping to rapidly restore ATP levels and remove excess succinate from tissues.

Succinate has been reported to strongly stimulate ROS generation due to the RET through mitochondrial Complex I^49^. This mechanism has been observed in mitochondria of mammals^30,48^ and reptiles^47,72^. However, in our previous study on *C. gigas*, we observed that RET does not contribute to the ROS efflux both under normal and short-term (15 min) H/R conditions^38^. Consistent with the notion of lack of RET, our present study found no increase in ROS efflux or FEL rate in succinate-energized oyster mitochondria after constant or cyclic H/R stress. There was also no accumulation of oxidative damage to proteins (indicated by protein carbonyls) after constant hypoxia in succinate-energized mitochondria. These findings indicate that a mild decrease in Complex II activity combined with a more significant suppression of Complex I effectively prevented RET and oxidative damage in oyster mitochondria under these conditions. However, after five H/R cycles a modest but statistically significant increase in the protein carbonylation was detected, indicating that the mitochondrial antioxidant systems might become overwhelmed under prolonged and frequent oxygen fluctuations.

## Conclusions and outlook

Mitochondrial bioenergetics of oysters is modulated by the constant hypoxia and cyclic oxygen fluctuations such as might occur during the shell closure and periodical valve gaping during the low tide. Mitochondrial Complex I appears to be the main target of H/R stress showing a gradual loss of activity with increasing duration of hypoxic exposure. There appears to be no major difference between the impact of constant and cyclic hypoxia of similar duration indicating that the mitochondrial damage is mostly determined by the lack of oxygen that leads to mitochondrial depolarization^73^. The damage to Complex I by H/R stress leads to a decrease in mitochondrial coupling efficiency and ATP synthesis capacity in oysters. In contrast to Complex I, Complex II-dependent succinate oxidation is considerably more robust to H/R stress showing only modest decrease after prolonged hypoxia (90–125 min, this study) and an increase after a short-term (15 min) hypoxia^27,74^. Combined with the lack of succinate-driven RET, highly robust succinate oxidation by oyster mitochondria might be considered an adaptive mechanism that permits flexible use of metabolic fuels and circumvents the limitations of Complex I during oxygen fluctuations.

Notably, the ROS efflux in the mitochondria of oysters was minimally affected by the H/R stress with no consistent evidence of the oxidative damage to mitochondria. This suggests that unlike the mitochondria of mammals where reoxygenation is associated with ROS burst^19,53,75,76^, mitochondria of stress-tolerant intertidal bivalves like oysters tightly control ROS efflux during environmental stress like oxygen (this study) and salinity^77^ fluctuations. In oysters, the robust forward electron flux with succinate combined with suppression of Complex I activity can minimize RET and stabilize ROS production in mitochondria during H/R stress. This aspect is particularly important in the intertidal species that often experience oxygen fluctuations from near anoxia to normoxia or even hyperoxia. Nevertheless, oxidative damage to proteins accumulates early if the respiration is fueled by NADH-linked substrates, and later after repeated H/R cycles with succinate. Further studies are needed to determine the possible functional consequences of mitochondrial protein carbonylation and determine the mechanisms responsible for the selective inactivation of Complex I by H/R stress in mitochondria of oysters and other stress-tolerant marine invertebrates.

## Data Availability

The datasets used and/or analyzed during the current study are available from the corresponding author on reasonable request.
